# Cellular Senescence in Cardiovascular Diseases: A Systematic Review

**DOI:** 10.14336/AD.2021.0927

**Published:** 2022-02-01

**Authors:** Can Hu, Xin Zhang, Teng Teng, Zhen-Guo Ma, Qi-Zhu Tang

**Affiliations:** ^1^Department of Cardiology, Renmin Hospital of Wuhan University, Wuhan 430060, China; ^2^Hubei Key Laboratory of Metabolic and Chronic Diseases, Wuhan 430060, China

**Keywords:** Cellular senescence, Cardiovascular diseases, Cardiac aging, Cardiomyocytes, Senotherapy

## Abstract

Aging is a prominent risk factor for cardiovascular diseases, which is the leading cause of death around the world. Recently, cellular senescence has received potential attention as a promising target in preventing cardiovascular diseases, including acute myocardial infarction, atherosclerosis, cardiac aging, pressure overload-induced hypertrophy, heart regeneration, hypertension, and abdominal aortic aneurysm. Here, we discuss the mechanisms underlying cellular senescence and describe the involvement of senescent cardiovascular cells (including cardiomyocytes, endothelial cells, vascular smooth muscle cells, fibroblasts/myofibroblasts and T cells) in age-related cardiovascular diseases. Then, we highlight the targets (SIRT1 and mTOR) that regulating cellular senescence in cardiovascular disorders. Furthermore, we review the evidence that senescent cells can exert both beneficial and detrimental implications in cardiovascular diseases on a context-dependent manner. Finally, we summarize the emerging pro-senescent or anti-senescent interventions and discuss their therapeutic potential in preventing cardiovascular diseases.

## 1.Introduction

Aging is a determinant risk factor for cardiovascular diseases, which is the leading cause of death around the world. Projections indicate that the incidence of cardiovascular diseases progressively increases with age, 1.3% for those aged between 55~64 years and 8.4% in those≥75 years of age [1,2]. The complex interactions between cardiovascular aging processes and various risk factors (e.g., hypertension, myocardial infarction, atherosclerosis and fibrosis) inevitably contribute to the development of heart failure. However, the mechanisms underlying the development of age-related cardiovascular diseases are not fully understood.

Cellular senescence is a state of stable cell-cycle arrest despite continued metabolic activity, which usually occurs in response to many endogenous and exogenous stresses during aging processes [3-5]. Historically, senescence was first identified by Hayflick and Moorhead half a century ago, who discovered that human diploid fibroblasts displayed a finite capacity for cell division because of telomere shortening (replicative senescence) [6]. Conversely, the telomere length-independent senescence was then observed in many aged or damaged tissues. Such stress-induced premature senescence (SIPS) can be triggered by distinctive stressful stimuli, including persistent DNA damage, oncogene activation, oxidative stress and mitochondrial dysfunction in cardiovascular system [7,8]. Eminently characterized by a proliferation arrest, the senescent cells are differed from other non-dividing cells (such as quiescent cells) with specific morphological and functional features. Growing evidences demonstrated that the senescent cardiovascular cells, including endothelial cells, vascular smooth muscle cells, fibroblast cells, cardiomyocytes, T cells and et al., were accumulated in the culprit lesions of cardiovascular system and act to improve or exacerbate the onset and outcome of cardiovascular diseases [9-11]. While cellular senescence imposes an important role in suppressing tumorigenesis. There is strong evidence that cellular senescence also participates in the progression of heart regeneration, cardiac remodeling, atherosclerosis and heart failure [5,12,13]. In this review, we first discuss the mechanisms and the features underlying cellular senescence. Then, we summarize the different types of senescent cells that present in cardiovascular systems and describe the pathophysiological implications of cellular senescence in cardiovascular disease. Moreover, we highlight the role of SIRT1 and mTOR in regulating senescence during age-related cardiovascular diseases. Finally, we focus on the emerging pro-senescent and anti-senescent therapies and discuss their therapeutic potential for cardiovascular diseases.

## 2.Molecular mechanisms of cellular senescence in cardiovascular system

**Figure 1.**
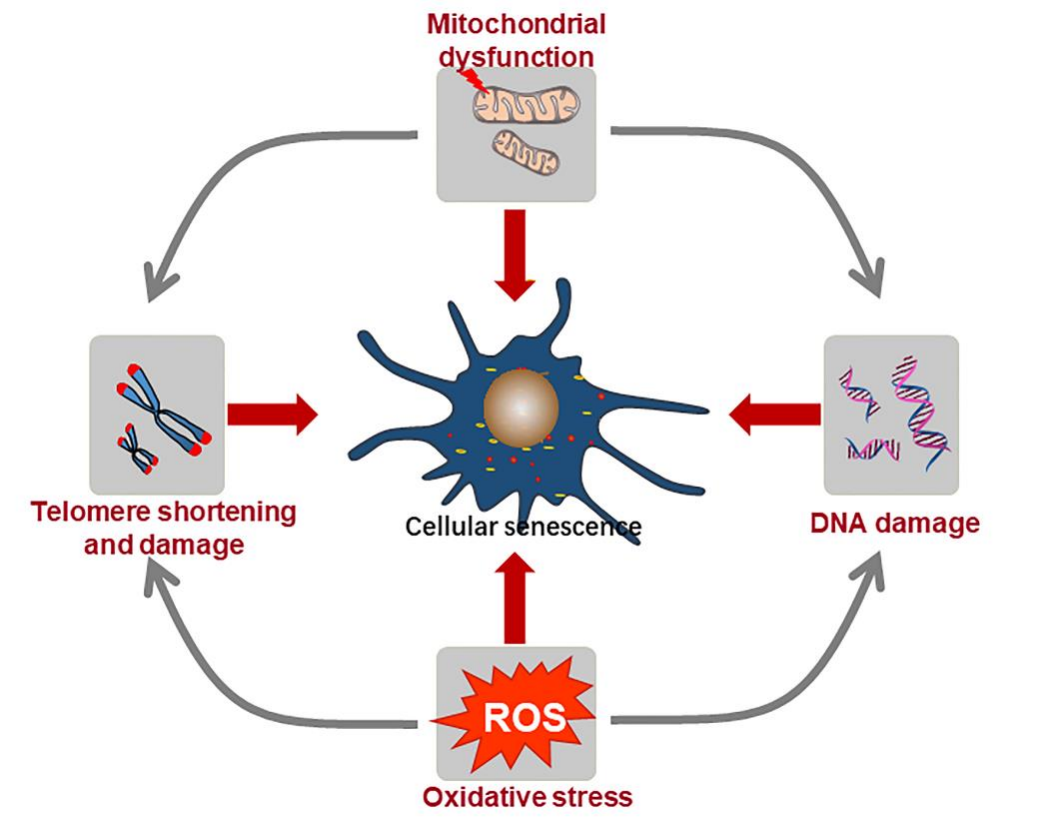
The mechanism in promoting cellular senescence in cardiovascular diseases. Telomere shortening, DNA damage, telomere damage, mitochondrial dysfunction and oxidative stresses can result in telomere length-dependent (replicative senescence) and length-independent (stress-induced premature senescence, SIPS) senescence in cardiovascular diseases.

Cellular senescence is a fundamental mechanism of various cardiovascular diseases. It is a stress or damage response that characterized by permanent proliferation arrest, tumor suppressor pathway activation, apoptosis resistance and special secretory phenotype in aged or diseased organisms [8]. By and large, two different kinds of cellular senescence have been studied during the progression of cardiomyopathy: replicative senescence and SIPS (Fig.1).

### 2.1 Replicative senescence

Replicative senescence, originally described by Hayflick, is a cell fate that cease to proliferate with finite division activities in vitro. The remarkable telomere shortening during consecutive cellular proliferation is one of the best-characterized mechanisms in replicative senescence [4]. Telomeres are special nucleoprotein complexes that located at the terminals of mammalian chromosomes to ensure chromosome stability during cell division. In general, telomere repeats decrease following each round of DNA replication as the standard DNA replication apparatus cannot duplicate chromosomal DNA ends in the absence of telomere maintenance mechanisms. Below a certain length, the loss of telomere-capping factors critically induces telomere DNA loop destabilization and contributes to chronic DNA-damage response (DDR) activation and irreversible G1 phase arrest [14]. Several observations have confirmed that telomeres shortening may reflect cellular proliferative ability throughout the lifespan. During aging, telomerase is an RNA-containing enzyme that plays a crucial role in maintaining telomere length to overcome senescence [15]. Therefore, the primary molecular events that responsible for replicative senescence may be telomere shortening and telomerase repression during biological aging.

Most of cardiovascular cells, including endothelial cells, VSMCs, fibroblast and immune cells, are mitotic cells with finite proliferative capacity. The progressive telomere loss-based senescence has been observed in heart tissues. For example, telomere length in cardiac tissues shortened with aging according to the real-time quantitative PCR analysis [16]. Murine endothelial cells exhibited age-dependent telomere shortening and telomerase repression in atherosclerotic arteries [17]. The evidence suggested a causal connection between telomere shortening, replicative senescence and cardiovascular diseases. According to longitudinal studies, telomere elongation was a potential therapeutic intervention in arresting replicative senescence. Overexpression of the telomerase component TRF2 has been shown to inhibit replicative arrest in VSMCs and promotes human plaque stabilization [20]. Therefore, telomerase may be a promising target in preventing telomere shortening and delaying age-related cardiovascular diseases during biological aging. Recently, considerable heterogeneity has been observed during the replicative senescence, which suggesting that replicative senescence was clearly associated with multiple stresses rather than a precise counting mechanism [18]. Indeed, telomere length is species specific and varies greatly upon different intrinsic and environmental stimulus. For example, VSMCs in atherosclerotic fibrous caps underwent a replicative senescence with telomere loss, which was further accelerated by oxidative stress [19]. Therefore, the majority of cardiovascular risk factors including oxidative stress, DNA damage and inflammation can modify telomere-shortening rate and accelerate replicative senescence in cardiovascular disorders.

### 2.1SIPS

In contrast to replicative senescence, SIPS is another type of cellular senescence that can be triggered by various external stimuli independent of telomere length, including persistent DNA damage, telomere dysfunction, mitochondrial dysfunction and oxidative stress. Here, we will first introduce these molecular mechanisms broadly and then discuss how these mechanisms mediate SIPS in cardiovascular system.

#### 2.2.1 Nuclear DNA damage

Cellular DNA can be damaged by numerous genotoxic factors during the lifespan, which elicits genome instability and transcription disorder inevitably. Substantial experimental evidence suggests that activation of DDR with DNA double strand breaks (DSBs) is a major determinant for age-associated pathophysiological changes and all stressors that result in persistent DNA damage can induce a senescent phenotype [21]. Mechanically, activation of DDR is usually accompanied with the formation of DNA damage foci, which is characterized by the phosphorylation of histone H2AX on Serine 139 at DSBs site [22]. Therefore, the DNA damage foci that stained positive for γ-H2AX is established as a reliable marker for persistent DNA damage and senescence.

In cardiovascular system, the genome suffers constant attack by various endogenous and exogenous genotoxic factors, including aging, blood flow, lipids, ischemia, et al. The VSMCs in advanced human atherosclerotic lesions showed extensive DNA damage and cellular senescent phenotypes [20]. Consistently, oxidized low-density lipoprotein could result in nuclear H2AX deposition and premature senescence in the murine aortic endothelium [23]. In other cardiac injury models, Cui observed increased senescent cardiomyocytes and DNA damage in myocardium postinfarction [24]. Therefore, DNA damage and consequence of the DNA damage (cellular senescence) are critically involved in the progress of cardiovascular disorders. To cope with the high frequency of DNA damage in DNA-based life, DNA damage repair has evolved as one of the best characterized mechanisms in promoting health and wellness. On the one hand, DNA damage repair can arrest cell cycle to avoid perpetuating damaged DNA into daughter cells. On the other hand, incomplete or incorrect DNA repair can also accelerate DNA damage and reinforce senescence by a large content. Recently, epigenetic alterations are reported to be associated with senescence by impinging on DNA damage repair process. Lyu et al observed that H4K20me3 reduction promoted cellular senescence during cardiac aging by compromising DNA damage repair and genomic maintenance [25]. Therefore, persistent DNA damage activation or DNA damage repair defects is a strong trigger to elicit premature senescence in cardiovascular diseases.

#### 2.2.2 Telomere damage

Telomeres are specialized nucleoprotein structures that are particularly sensitive to stresses during the aging processes. Hewitt demonstrated that telomeres were favored target for DDR and half of the DNA damage foci were collocated at telomeres in stress-induced senescence irrespective of telomerase activity [26]. Therefore, the presence of DNA-damage foci that located at telomere region is a hallmark of cellular senescence, which is described as telomere-associated foci (TAF). Detection of TAFs at telomeres provides direct evidence to quantify telomere damage in response to stresses. On the one hand, the co-localization analyses of DDR factors (including 53BP1 and γ-H2AX) and telomere repeats are applied to verify TAF typically. Fumagalli et al performed fluorescence in situ hybridization (FISH) with a telomere Cy3-conjugated peptide-nucleic acid probe (PNA) and immunofluorescence staining against 53BP1 to identify the presence of TAF in fibroblasts [27]. This dual immune-FISH staining method suggested a co-localization between 53BP1 and telomeres in senescent fibroblasts after ionizing radiations. In addition, dual staining of DNA damage foci (53BP1) and telemetric-repeat binding factor (TRF) also revealed telemetric foci in somatic cells [28]. TRFs are important components of the shelterin complex that are essential for maintaining chromosome stability. De-protection of telomere by TRF2 depletion was sufficient to elicit growth arrest and premature senescence in human and murine cells [20]. Therefore, TRF2 has been implicated in premature senescence via regulating telomere dysfunction.

Surprisingly, the theory of telomere length-independent telomere damage provids a novel insight into investigating cellular senescence in postmitotic cardiomyocytes. Anderson initially observed both telomeric DNA and non-telomeric DNA damage in cardiac myocytes with H_2_O_2_ treatment and X-ray irradiation [29]. While only telomeric DNA damage was persistent and irreparable over a long-term course of irradiation. More importantly, specific induction of TAF by TRF1-Fok1 fusion protein elicited cellular senescence in cardiomyocytes, occurring irrespectively of telomere length [29]. This suggested that persistent DNA damage at telomere could induce premature senescence in cardiomyocytes. To further elucidate the connection between telomere damage and cellular senescence in cardiovascular diseases, gain-of and loss-of function studies of TRF2 was applied in plaque VSMCs in atherosclerosis [20]. Along with decreased TRF2 expression, VSMCs in advanced human plaques were characterized by increased telomere damage and cellular senescence. Significantly, TRF2 overexpression accelerated DNA repair and bypassed premature senescence in atherosclerotic plaque VSMC [20]. Thus, telomeres are critical targets for stressful insult and telomere DNA damage is a frequent component of SIPS in cardiovascular diseases.

#### 2.2.3 Mitochondrial dysfunction

In the context of aging, abnormalities in mitochondrial structure and function are regarded as major contributing factors to age-related dysfunction [30-33]. Indeed, both mitochondrial dysfunction and cellular senescence are considered as key hallmarks of pathological aging. Recently, emerging evidence has begun to uncover the intricate relationships between the mitochondrial dysfunction and cellular senescence [34]. One of the best characterized mechanisms by which mitochondrial dysfunction elicits senescence is the excessive by-product production of ROS during respiration [35]. Additionally, other mitochondrial mechanisms, including mitochondrial calcium homeostasis, mitochondrial quality control mechanism and mitochondrial dynamics, also participate in establishing premature senescence [35,36].

Cardiac myocytes are abundant in mitochondria for their high-energy demands and are particularly susceptible to mitochondrial dysfunction. A recent study supported the concept that increased mitochondrial ROS production could result in senescent phenotypes in aged heart [29]. The cardiomyocytes isolated from old mice exhibit markedly mitochondrial dysfunction, as demonstrated with disturbed electron transport chain and decreased mitochondrial proteins [29]. To elucidate the exact role of mitochondrial dysfunction in cellular senescence. A transgenic mouse model of cardiomyocytes-specific overexpression of MAO-A was generated, which displayed enhanced mitochondrial ROS [29]. Interestingly, an increased telomere dysfunction was observed in these mice, along with increased expression of TAF and senescent marker in cardiomyocytes. Therefore, Anderson concluded that age-dependent mitochondrial dysfunction could contribute to cellular senescence in cardiomyocytes via irreparable telomere damage [29]. Previously, intervention that repressing mitochondrial ROS demonstrated reduced DNA breaks at telomere regions and extended the lifespan during aging. In vivo, L5 augmented mitochondrial oxygen consumption and mitochondrial ROS in the aortic endothelium, as well as increased nuclear γ-H2AX foci and SA-β-gal activity [23]. Significantly, co-administration of n-acetyl cystenine (NAC, an antioxidant) alleviated L5-induced mitochondrial ROS and senescent biomarkers in mice. More importantly, L5-induced increase in nuclear γ-H2AX deposition and SA-β-gal activity were also attenuated by NAC in human aortic endothelial cells [23]. In brief, these results suggested that L5-induced endothelial senescence were mitochondrial ROS-dependent and reinforced the interpretation that mitochondrial dysfunction was an important contributor to cellular senescence in cardiovascular diseases.

#### 2.2.4 Oxidative stress

The free-radical theory of aging was initially proposed by Harman half century ago, which suggested that accumulation of oxygen radicals should be the leading driving forces for age-related pathological disorders [37]. In accordance with this hypothesis, several studies revealed that oxidative damages were responsible for lifespan limitation and antioxidants exhibited a prolonged lifespan [38,39]. Indeed, oxidative stress is known to promote protein peroxidation, telomere shortening and DNA damage, all of which have been implicated in DDR and senescence [40-42]. Senescence, in turn, contributes to inflammatory response and ROS generation via the SASP, which further reinforce the activation of cellular senescence [43]. Therefore, the connection between oxidative stress and cellular senescence are complex and not fully understood.

In cardiovascular systems, mitochondrial oxidative metabolism is the pivotal intracellular mechanism of oxidative stress throughout the life. As described above, the mitochondrial-ROS can accelerate DNA damage and eventually lead to premature senescence in cardiovascular diseases [29]. Targeting oxidative stress on which senescent cells depend was reported to prevent cellular senescence and promote health aging. Glutathione reductase (GR) is a flavoprotein oxidoreductase, which sustain the antioxidant capacity of cells by regulating the GSH/GSSG ratio. A recent study by Chen found that GR repression was involved in Klotho-deficiency-related cardiac aging and cardiomyocytes senescence [44]. Cardiac-specific overexpression of GR prevented oxidative stress and rescued senescence marker (p16) levels in Klotho-knockout mice. Collectively, oxidative stress plays an important role in cardiac aging and targeting oxidative stress is a therapeutic strategy for preventing cardiac aging.

## 3Evidence of cellular senescence in cardiovascular diseases

Given the complex intracellular environment, identification of senescent cells in cardiovascular system should be challenging and meaningful. Therefore, we first discuss the senescence biomarkers in cardiovascular system broadly. Then, we will review the existence of different senescent types in cardiovascular diseases by combining different senescence biomarkers.

**Figure 2.**
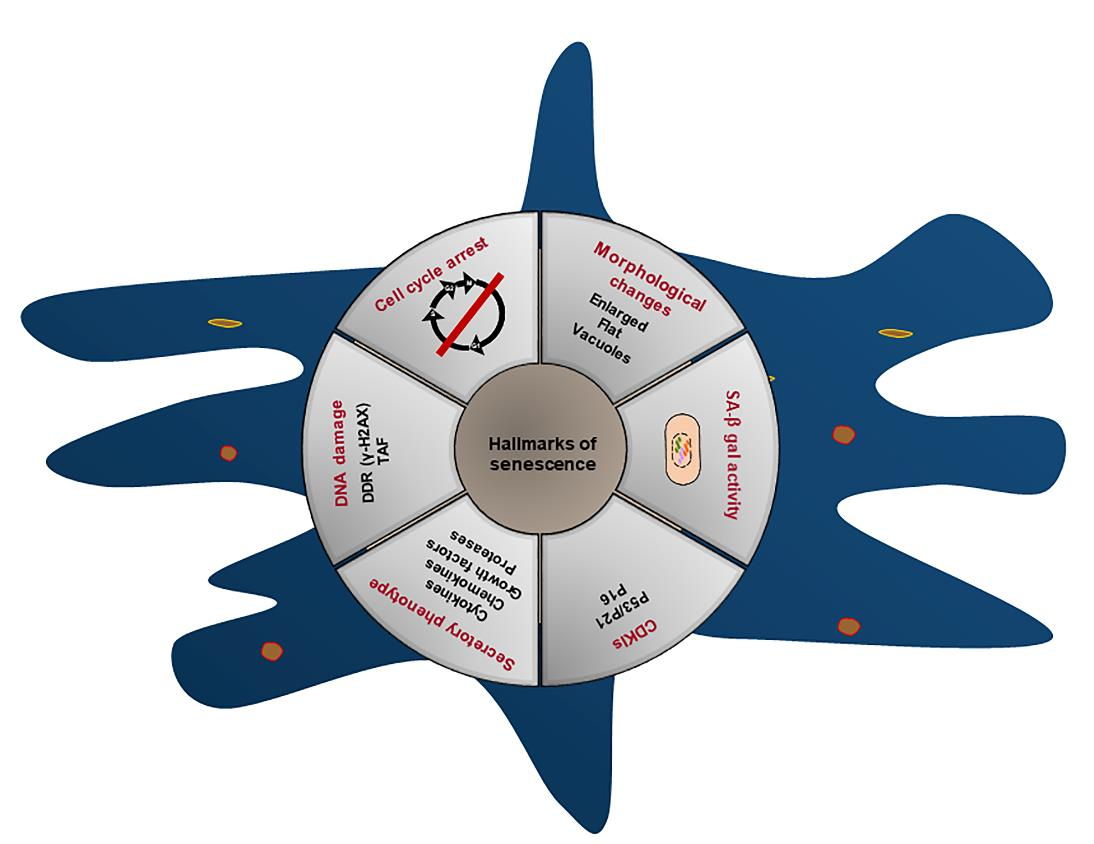
The hallmarks of senescent cells. (1) Irreversible cell cycle arrest. (2) Senescent cells display a characteristic enlarged and flatten morphological changes. (3) Increased senescence-associated β-galactosidase (SA-β-gal) activity. (4) Permanent cell-cycle withdrawal requires the expression of cyclin-dependent kinase inhibitors (CDKIs), most notably the P21 and p16. (5) Activation of the senescence-associated secretory phenotype (SASP), a bioactive secretome containing cytokines, chemokines, growth factors, proteases and other signaling molecules. (6) DNA damage response (DDR) (including DNA damage in telomere regions).

### 3.1 Markers of cellular senescence in cardiovascular diseases

Phenotypically, the senescent cells are heterogeneous and apparent with a wide variety of features and markers at the morphological and molecular levels (Fig. 2). Yet, there is no consensus on a single senescence biomarker that can specifically applied to identify senescence. A more in-depth understanding of the features and markers of cellular senescence is absolutely urgent.

#### 3.1.1 Morphological features

A defining feature of cellular senescence is the distinct morphological changes during progression. J. Ball indicated that the senescent fetal cardiomyocytes were invariably characterized with enlargement and vacuolization cell body [45]. Such morphological change in cardiomyocytes was one of the first indication of senescence after hypoxia treatment. Doxorubicin is a common anticancer agent with inevitable cardiotoxicity by disturbing DNA integrity. Therefore, doxorubicin is an established senescence stressor and the neonatal rat cardiomyocytes treated with doxorubicin showed enlarged volume, flattened morphology and vacuoles, similar to the morphological changes in aged cardiomyocytes [46]. Surprisingly, the morphological characteristics are cell-type independent during cardiac aging. The enlarged and flattened senescent phenotypes were also observed in senescent human umbilical vein endothelial cells (HUVECs) by Sirt1 inhibition [47]. Moreover, VSMCs from atherosclerotic plaque also exhibited large and flattened morphological features with growth arrest [48]. Thus, morphological alterations may facilitate the identification of senescent cells in cardiovascular diseases despite the lack of cell-type specifity.

**Figure 3.**
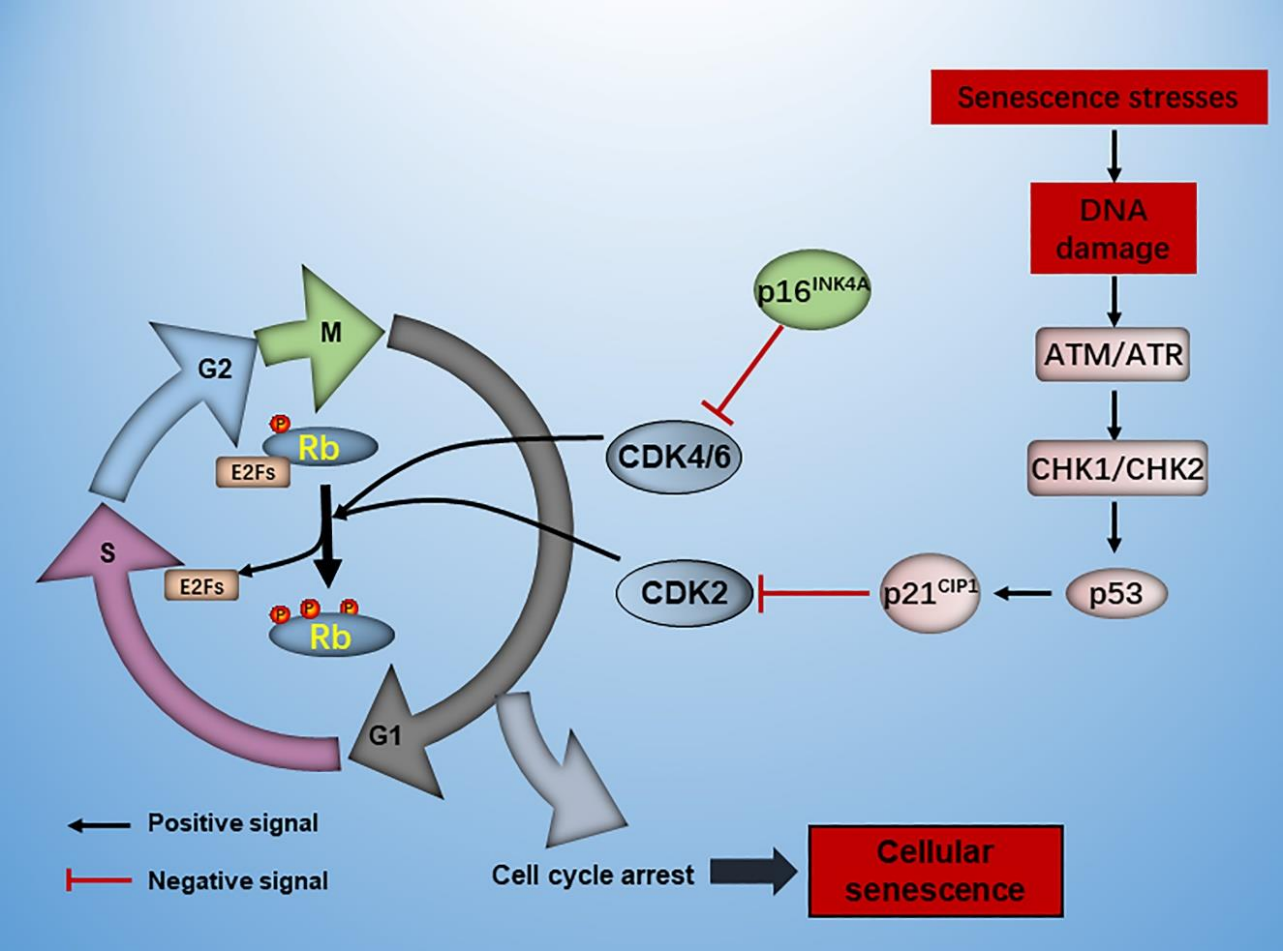
The pathways in regulating cell cycle arrest. The multiple stresses activate DNA damage response (DDR), which directly activates p53 and its downstream target p21 via the kinase cascades involving apical kinases ataxia telangiectasia mutated (ATM), ATR and the downstream kinases CHK2 and CHK1. P21 can inhibit the activation of CDK2. P16 can increase during stressful insult and inhibit CDK4/6. Together, the CDKIs (p16 and p53/p21) inhibit CDK2/4/6 and then suppress the phosphorylation of retinoblastoma protein (Rb). The nonphophorylated Rb inhibit the transcriptional activity of E2F and result in cell cycle arrest.

#### 3.1.2 Molecular markers

##### 3.1.2.1 p53 tumor suppressor pathway

Cellular senescence is an essential tumor-suppressive mechanism that prevents damaged cells from neoplastic proliferation. A variety of experiments discussed the involvement of p53 tumor suppressor pathway in senescence and tumor suppression. Persistent genotoxic stressors trigger DNA damage and activate DDR, which resulting in increased deposition of γ-H2AX and 53BP1 in chromatin [49]. These stressful signals subsequently activate p53 tumor suppressor pathway via the kinase cascades involving apical kinases ataxia telangiectasia mutated (ATM), ATR and the downstream kinases CHK2 and CHK1 (Fig. 3). In the context of DNA damage, activated p53 transcriptionally regulates the downstream genes that involved in cell cycle arrest and senescence. Therefore, tumor suppressor p53 is a decision-making transcription factor that integrates diverse cell fate, especially cellular senescence.

Recent data have underscored the involvement of p53-dependent senescence in cardiovascular system. As an essential senescence regulator, tumor suppressor p53 expression was increased in the infarcted heart or hypoxia-treated fibroblast [50]. Importantly, gain-of and loss-of function of p53 was performed to determine the pivotal role of p53 on fibroblast senescence. Overexpression of p53 exacerbated senescence in fibroblasts, whereas of which depletion abolished senescence. Generally, p53 protein is regulated by diverse posttranscriptional modifications. Sarig et al identified a transient increased expression of p53-S23 in neonatal heart post resection [51]. In adult mice, p53-S23 expression was dramatically increased in agrin-treated heart. Those results confirmed that cellular senescence is associated with activation of p53 tumor protein, which serves as a biomarker of senescence in cardiovascular diseases.

##### 3.1.2.2 CDKIs

Mammalian cell cycle is a highly conserved biological process that controlled by cyclin, cyclin dependent kinases (CDKs) and cyclin dependent kinase inhibitors (CDKIs) [52]. In general, cyclin interacts with CDKs to promote cell cycle progression. Under genotoxic stress conditions, the CDKIs, including INK4 and CIP/KIP, target to CDK2 and CDK4/6 and result in cell cycle withdrawal (Fig. 3). Precisely, the members of INK4 family consist of p15^INK4^, p16^INK4^, p18^INK4^ and p19^INK4A^. Whereas the CIP/KIP family include p21^CIP1^, p27 ^KIP1^ and p57^KIP2^.

In mammal cells, permanent cell-cycle withdrawal is typically regulated through two main pathways, p53/p21^CIP1^ and p16^INK4A^/Rb, which have been widely used as biomarkers to identify cellular senescence in vivo and in vitro. p21, encoded by *CDKN1*α, is a downstream mediator of p53 pathway that targets CDK2 to initiate senescence in response to stressors [53]. In murine and human senescent cells, the p21 expression was increased and elicited cell cycle arrest at G1 phase. The upregulation of p21 expression was then analyzed as a senescence biomarker in response to different stressors (Table 1). Recently, various studies argued that p21 was not the best-recognized marker for cellular senescence, as the activation of p21 in senescent cells does not persist and decreases after the establishment of growth arrest.

p16 is an Rb-regulated CDKI that plays a prominent role in maintaining the senescent phenotype by inhibiting CDK4/6, Rb phosphorylation and E2Fs activity. In aged diseased heart, p16 was increased and nearly 50% of cardiac myocytes were p16-positive [54]. Therefore, p16 is a predominant marker for cellular senescence and age-related accumulation of p16-positive cells negatively impacts longevity [55]. Recently, great progresses have been made in the engineered mouse models (INK-ATTAC and p16-3MR) in which p16-positive senescent cells can be selectively eradicated. Clearance of p16-positive senescent cells delayed tumor progression and age-dependent disorders in multiple tissues and organs, including heart [55]. Therefore, p16 is one of the best-studied biomarkers during senescence. Other CDKIs, including p27, p21 and p15, also accumulated in senescent cells and were essential for maintaining cell cycle arrest [8]. Despite the fact that detection of CDKIs expression is a convenient method in exploring senescence. It needs to be noted that the expression of these molecules does not provide conclusive evidence, but need combination of various biomarkers.

**Table 1:** The role of senescent cardiovascular system cells in cardiovascular diseases.

Cell types	Ref	model	Stressors/diseases	Hallmarks of senescence	Pathological effect	Effects
Cardiomyocytes (CMs)	[25]	In vitro	Isolated adult CMs	SA-β-gal activity	Age-related myocardial dysfunction	Detrimental
	[62]	In vitro	H/R	Cell proliferation, SA-β-gal, p16, telomerase	N	N
	[64]	In vitro	Doxorubicin	SA-β-gal activity, TRF1/2, P53	N	N
	[63]	In vitro	Doxorubicin	SA-β-gal activity, telomerase activity, p53/p21, p16, p27	N	N
	[87]	In vivo	Doxorubicin	SA-β-gal activity, p16,	Doxorubicin-related cardiotoxicity	Detrimental
		In vitro				
	[157]	In vivo	Aged mice	Telomere length, γ-H2AX, p53, P21, SA-β-gal activity	Age-related myocardial dysfunction	Detrimental
		In vitro	H2O2			
	[24]	In vivo	MI	p16, p21, SA-β-gal activity, SASP	Anti-fibrosis and improves heart dysfunction	Beneficial
		In vitro	Hypoxia			
	[29]	In vivo	Aged mice	Cell proliferation, Telomere length, γ-H2AX, TAF, telomerase activity, p21, SASP	Age-related myocardial dysfunction	Detrimental
		In vitro	Isolated adult CMs			
Endothelial cells (ECs)	[67]	In vivo	SAM	P53/CD31 co-staining, SA-β-gal activity	Age-related Heart failure with preserved EF	Detrimental
	[47]	In vitro	Sirt1 inhibition	Cell proliferation, morphology, SA-β-gal activity, p53	N	N
	[158]	In vitro	High glucose	SA-β-gal activity, p53, p21, p16	Oxidative stress and endothelial dysfunction	Detrimental
	[66]	In vivo	Aged rat	SA-β-gal activity, p21, p16	Anthocyanins protect against endothelial senescence	Detrimental
		In vitro	D-galactose			
	[17]	In vivo	Atherosclerosis	SA-β-gal activity, p16, p21	Decreased eNOS activity, endothelial dysfunction	Detrimental
		In vitro	TRF2 inhibition			
	[159]	In vivo	PAH rat	SASP, p16, p21, γ-H2AX	Irreversible PAH	Detrimental
		In vitro	Isolated pMVECs			
	[23]	In vivo	L5 injected mice	γ-H2AX, SA-β-gal activity, ATM, Chk2, p53	Atherosclerotic vascular disease	Detrimental
		In vitro	L5 treated HAECs			
	[100]	In vivo	Human thoracic aorta	Cell proliferation, SA-β-gal activity, telomerase activity	Progression of atherosclerosis	Detrimental
		In vitro	High glucose			
Vascular smooth muscle cells (VSMCs)	[48]	In vitro	Ras activation	Cell morphology, SA-β-gal activity, p53, p21, p16	Inflammation and atherosclerosis	Detrimental
	[160]	In vivo	Atherosclerosis	P53, p21, p16, SASP	Inflammation and plaques progression	Detrimental
	[78]	In vitro	AngII	SA-β-gal activity, p16, p21	N	N
	[74]	In vitro	AngII	SA-β-gal activity, p16, p21, p53, p27	Oxidative stress and age-related vascular disorder	Detrimental
	[161]	In vivo	AngII	γ-H2AX, p16, p21, SA-β-gal activity	Vascular remodeling and senescence	Detrimental
	[162]	In vitro	IL-1β	Telomerase activity, SA-β-gal activity, p16, p21	N	N
	[163]	In vitro	Isolated aged VSMCs	SA-β-gal activity, p53, p21	N	N
	[164]	In vitro	Aging VSMCs (p15)	γ-H2AX, SA-β-gal activity	N	N
	[101]	In vivo	Atherosclerosis	SA-β-gal activity, p16, γ-H2AX	Inflammation and atherosclerosis	Detrimental
		In vitro	Isolated VSMCs			
	[20]	In vivo	Atherosclerosis	γ-H2AX, ATM, p16,p53, TRF2	Atherosclerosis and plaque vulnerability	Detrimental
		In vitro	Isolated VSMCs			
	[19]	In vivo	Human plaques	SA-β-gal activity, p16, p21, telomere length, cell Proliferation	Atherosclerosis	Detrimental
		In vitro	Isolated VSMCs			
	[165]	In vivo	Hypertension	γ-H2AX, p16, p21, CD4K, SA-β-gal activity	N	N
		In vitro	AngII			
	[102]	In vivo	Calcified arteries	SA-β-gal activity, p16, p21, p53, γ-H2AX, ATM, proliferative capacity	Vascular inflammation and calcification	detrimental
		In vitro	p14-15 VSMCs			
Fibroblasts/ Myofibroblasts	[51]	In vivo	Resection of hearts	SA-β-gal activity, p53	Promote cardiac regeneration	Beneficial
	[81]	In vivo	Apical resection	SA-β-gal activity, SASP, cell proliferation	Promote cardiac regeneration	Beneficial
	[105]	In vivo	Aging mice	SA-β-gal activity, p53, p21, p16, cell proliferation	Restrict cardiac aging and fibrosis	Beneficial
	[58]	In vivo	TAC	SA-β-gal activity, p21, p16, p53	Restrict myocardial fibrosis and improve cardiac function	Beneficial
		In vitro	CCN1 activation			
	[50]	In vivo	MI	SA-β-gal activity, p53, p21, p16, p19, cell morphology	Heart rupture after infarction	Detrimental
		In vitro	H/R			
	[98]	In vivo	MI	Cell proliferation, SA-β-gal activity, γ-H2AX, p21	Limit cardiac fibrosis and remodeling post MI	Beneficial
		In vitro	Ionizing radiation			
	[80]	In vivo	MI	P21, p19, p53, p16, SA-β-gal activity,	Promote angiogenesis and limit cardiac remodeling post MI	Beneficial
		In vitro	Hypoxia/oxygenation			
T cells	[94]	In vivo	MI	Flow cytometry, CD8^+^CD57^+^ T cells	Inflammation and cardiovascular mortality	Detrimental
	[92]	In vivo	Peripheral blood	Flow cytometry, CD4^+^CD57^+^ T cells	Arterial Stiffness	Detrimental
		In vitro	Cytomegalovirus			
	[87]	In vivo	CHD	Flow cytometry-FISH, CD8^+^CD28^-^ T cells	Increased inflammation and CHD	Detrimental
	[90]	In vivo	Acute heart failure	Flow cytometry, CD4^+^CD57^+^ T cells	Pro-inflammatory features	Detrimental
	[93]	In vivo	Hypertension	Flow cytometry, CD4^+^CD57^+^ or CD28^-^T cells	Pro-inflammatory features	Detrimental

TRF, telomere binding factor; MI, myocardial infarction; H/R, hypoxia reoxygenation; SAM, senescence accelerated mice; PAH, pulmonary arterial hypertension; pMVECs, pulmonary microvascular ECs; p15, Passage 15; TAC, Transverse aortic constriction; HAECs, human aortic endothelial cells; AngII, angiotensin II; CHD, coronary heart diseases; EF, ejection fraction; N, not mentioned.

##### 3.1.2.3 Senescence associated-β-galactosidase (SA-β-gal) activity

One of the best characterized features for senescence is the positive staining for SA-β-gal at PH6, which indicates a marked expansion of lysosomal compartment in functional-altered senescent cells [56]. SA-β-gal staining was first applied to recognize senescent fibroblast in aging skin and is still the most widely used markers to detect senescent cells nowadays [57]. With progression, the SA-β-gal activity has become an important instrument in detecting the location of senescent cells in aged or diseases tissues. Co-immunostaining of SA-β-gal and specific cell markers, such as cardiomyocytes-specific markers for troponin T or α-actinin, myofibroblast-specific marker for α-smooth muscle actin (α-SMA), endothelial cell-specific marker CD31 and fibroblast marker vimentin, was used to identify senescent cell types in cardiovascular diseases. For example, SA-β-gal-positive myofibroblasts were accumulated in the perivascular fibrotic areas of transverse aortic constriction-treated mice [58]. By co-labelling with troponin-C, Anderson et al detected increased SA-β-gal activity in cardiomyocytes in aged mice [29]. Recently, an arguing comment about the SA-β-gal activity is that this marker is also detectable in cells under serum-starve or lysosome-enriched macrophages [59]. Therefore, developing a more convenient, accurate detection commercial kit of SA-β-gal activity is promising in the future.

##### 3.1.2.4 Senescence associated secretory phenotype (SASP)

It has long been discussed that the cultured medium of senescent cells is enriched with numerous secreted proteins. Initially, Campisi et al observed that matrix metalloproteinase 3 (MMP3), secreted by senescent fibroblasts, could promote the transformation of pre-malignant-mammary epithelial cells [60]. This observation suggested that senescence was an active process with diverse properties, but not a mere passive process with permanent state of cell cycle arrest. And proposed that SASP was the primary mechanism to mediate the biological functions of senescence by secreting inflammatory cytokines, chemokines, growth factors and proteases.

Considering the fact that most of senescent cells is accompanied by a marked activation of SASP. We tend to discuss the implication of SASP in cardiovascular diseases. For example, the mRNA levels of Il-6, Il-11, Cxcl1, CxclL2 and M-CSF were increased in hypoxia-treated fibroblasts [50]. Depression of senescence by p53 deficiency markedly reduced the secretory senescence factors in cardiac fibroblasts and in infarcted heart, accompanied with decreased collagen deposition and cardiac fibrosis [50]. Accordingly, SASP activation is a dynamic, cell type-dependent process that can influence the surrounding cellular microenvironments and drive organismal disorder. Anderson analyzed the inflammatory SASP factors, including Il-1a, Il-1b, Il-6, Cxcl1 and Cxcl2, in purified senescent cardiomyocytes by RNA-sequence [29]. Surprisingly, they found a significant difference in the expression of SASP components between purified cardiomyocytes and whole heart homogenates. The purified senescent cardiomyocytes failed to increase the expression of Il-6 and Cxcl1. Rather, it activated a non-canonical SASP with increased Edn3, Tgfb2, and Gdf15 expression to promote cardiac hypertrophy. Therefore, the composition and function of SASP secreted in cardiovascular systems differ in a context-dependent manner and the SASP complexes not only include pro-inflammatory cytokines but also molecules involved in cardiac remodeling.

### 3.2 Presence of senescent cells in cardiovascular diseases

The senescent cells that accumulated in cardiovascular diseases are discussed as follows, including cardiomyocytes, endothelial cells, VSMCs, fibroblasts/ myofibroblasts and T cells. In this section, we will review the senescence-related mechanisms and biomarkers in different cardiac cell types to demonstrate the presence of senescence in different pathological models.

#### 3.2.1Cardiomyocytes senescence

Cellular senescence is traditionally recognized as a state of irreversible G1 arrest in mitotic cells. However, the majority of cardiomyocytes are terminally differentiated cells, withdrawing from the cell cycle immediately after birth. Despite argued for decades, accumulating body of research indicated that cardiomyocytes also equipped with mechanisms to drive senescence.

The mammalian heart is an obligate aerobic organ that struggling to deliver oxygen for energy metabolism after birth. With aging, the myocytes exhibit impaired metabolic flexibility and excessive mitochondrial ROS production, which resulting in DNA damage and telomere dysregulation [29]. Therefore, there is an age-dependent increase of senescent cardiomyocytes in murine heart [29]. The cardiomyocytes isolated from old mice have a remarkable senescence-like phenotype, including hypertrophy, classical senescent growth arrest pathways and increased SA-β-gal staining [61]. In addition to biological aging, senescent cardiomyocytes are also implicated in multiple pathological disorders, including myocardial ischemic damage, diabetic cardiomyopathy and doxorubicin-induced cardiomyopathy et al. Zhang determined the presence of cardiomyocytes senescence in acute myocardial infarction cardiac tissues [62]. In vitro, he found increased senescence biomarkers in neonatal rat cardiomyocytes under hypoxia reoxygenation, including P16 mRNA levels and SA-β-gal activity. Although lack of specific biomarkers to identify senescent cardiomyocytes in vivo, dual immunostaining of p16 (a senescence biomarker) and α-actinin (a cardiomyocytes biomarker) confirmed increased localization of senescent cardiomyocytes in postinfarction murine heart [24].

Doxorubicin is a potent antitumor agent with inevitable DNA damage in mammalian cells. The linkage between doxorubicin and cardiomyocytes senescence was examined follow with great interest. As expected, Maejima found that low dose of doxorubicin (10^-7^mol/L) could elicit senescence in neonatal rat cardiomyocytes, as characterized with morphologically flattened and enlarged cell shapes and classical senescent biomarkers [63]. Consistently, the H9C2 cardiomyocytes could reproduce senescence after 0.1μM of doxorubicin treatment, with increased senescence markers (p21 and p16) and pro-inflammatory SASP [64]. Yet, very little is known about the mechanisms underlying doxorubicin-related cardiomyocytes senescence. Doses of stressors likely contribute to different cell fate between senescence and apoptosis. Spallarossa et al treated cardiomyocytes with various doses of doxorubicin and found that low doses of doxorubicin triggered senescence, while high doses induced apoptosis via regulating TRF1 and TRF2 [64]. Therefore, cardiomyocytes senescence is a complex mechanism that involved in both healthy cardiac aging and divers heart diseases. Further depth understanding of cardiomyocytes senescence will be helpful to develop novel senescence models and investigate promising therapeutic approaches for senescence-associated cardiac diseases.

#### 3.2.2Endothelial cells (ECs) senescence

ECs form a protective barrier in the inner layer of blood vessels, of which dysregulation is closely correlated with endothelial dysfunction and aortic disorders. As with most other mammalian cells, the proliferative capacity of ECs is limited with age. Previously, Augustin-Voss et al investigated the phenotypic changes, migratory and proliferative alterations of bovine aortic endothelial cells (BAECs) during long-term culture [65]. They found a steady decline of migration and proliferation rates during BAECs senescence in vitro culture. Morphologically, the senescent BAECs (passages 35-45) formed a dense cobblestone-like, growth-arrested monolayer, but lacked crisscrossing morphotype in senescent cultures. This study represented a phenomenon of ECs senescence after long-term population doublings. In vivo, the senescent ECs were accumulated throughout the aortic wall of aging rats compared with young groups and contributed to age-related endothelial dysfunction [66]. The senescence-accelerated mice (SAM) is an aging model that displaying early endothelial dysfunction and heart failure. By dual immunostaining with CD31, Gevaert observed increased acetyl-p53 expression in endothelial cells in the injured aortic segments of SAM [67]. This coincided with increased endothelial inflammation and arterial stiffness. Therefore, age-related endothelial dysfunction is probably driven by the accumulation of senescent ECs and pro-inflammatory SASP.

The dysregulation of plasma lipids is a pivotal mechanism of endothelial dysfunction and atherogenic response. In this regard, Wang et al investigated the mechanisms underlying vascular senescence and L5 in human aortic endothelial cells (HAECs) [23]. They found that L5 elicited DDR and cellular senescence in cultured HAECs, accompanied with increased γ-H2AX and SA-β-gal activity. This suggested that senescence was involved in dyslipidemic-related vascular disorders during progression. The major risk factors for atherosclerosis include lipid disturbance and endothelial dysfunction. In dyslipidemic *LDLr^-/-^;hApoB100^-/-^* atherosclerotic mice, p21 expression was increased in the endothelial of the aorta but not altered in the media, suggesting accumulated senescent ECs in murine atherosclerotic lesion [68]. More importantly, vascular ECs with senescent phenotypes were also reported in human atherosclerotic arteries. Minamino et al found increased SA-β-gal-positive cells near the luminal surface (mainly composed with endothelial cells) of human coronary arteries [17]. Immunohistochemistry staining of factor VIII (an EC biomarker) confirmed that SA-β-gal-positive cells, which predominately localized on surface of atherosclerotic plaques, were vascular ECs. Despite these advances, the implication of endothelial senescence on microvascular network remains under-investigated. Recent studies reported that senescent ECs were accumulated in the aorta of diabetic rats, and exposure of ECs to high glucose (25mmol/L) elicited senescence phenotypes with increased SA-β-gal activity [69]. Therefore, ECs senescence may also participate in diabetes-related microvascular complications, including ischemic heart diseases and cerebrovascular disorders. Interventions and therapies targeting ECs senescence would have important clinical implications.

#### 3.2.3VSMCs senescence

VSMCs are major cell types that present at the medial layer of blood vessel to maintain vascular homeostasis. Studies indicate that VSMCs can migrate into the intima and shift from a contractile to a synthetic phenotype, which is essential for vessels remodeling and atherosclerotic plaque stability [70]. Like ECs, VSMCs were susceptible to different vessel stressors, including DNA damage, inflammation, oxidative stress and angiotensin II (Ang II), and developed senescence phenotypes correspondingly. Aged vessels and advanced atherosclerotic lesions showed abundance of SA-β-gal-positive VSMCs, which were characterized by reduced proliferative capacity and extensive DNA damage [71]. Specifically, the occurrence of VSMCs senescence in atherosclerosis has been reviewed previously (as reviewed in Ref [70,72]). VSMCs isolated from human atherosclerotic plaques underwent a stable growth arrest and showed increased hypo-phosphorylated RB and p53 expression compared to normal groups [73]. In addition to atherosclerosis, recent studies have linked VSMCs senescence to other vascular aging-related disorders, including abdominal aortic aneurysm (AAA), hypertension and diabetes. Here, we will discuss presence of VSMCs senescence in these diseases.

AngII is a potent systemic vasoconstrictor that involved in various vascular pathological processes by accelerating VSMCs senescence. Indeed, the SA-β-gal activity was increased in human VSCMs with chronic AngII stimulation [74]. AAA is the most common aortic aneurysm occurring in humans. In AngII-induced AAA models, the medial VSMCs proliferation capacity was decreased, and the senescence biomarkers were significantly increased in mice aortas [75-77]. Mechanically, AngII binds angiotensin receptor and activates the renin-angiotensin system (RAS) to regulate vessel constriction. Such systemic RAS activation is correlated with increased pro-inflammatory cytokines and free radicals in VSMCs. Therefore, Tsai et al investigated the mechanisms underlying AngII-related VSMC senescence [78]. They found that AngII stimulated ROS generation and senescent phenotypes in human aortic VSMCs, which were alleviated by ROS scavengers and membrane-permeable catalase. These results suggested that AngII elicited VSMCs senescence via accelerating ROS production. Generally, VSMCs coordinated with ECs to participate in macrovascular complication in diabetes. However, whether high glucose causes VSMCs senescence has rarely been reported. Cui et al demonstrated that high glucose elicited significant senescent phenotypes in human VSMCs, as verified by an increased expression of SA-β-gal activity and cell cycle inhibitors [79]. More importantly, the senescence biomarkers were elevated in saphenous vein-SMCs that isolated from type 2 diabetes patients. The diabetic-derived VSMCs exhibited increased SA-β-gal activities and SASP expression, as well as impaired proliferation and augmented DNA damage. Collectively, senescent VSMCs are located in various vascular injuries and the clinical consequence of VSMCs senescence will be discussed later.

#### 3.2.4 Fibroblast and myofibroblast senescence

Originally, L. Hayflick et al discovered a limited proliferation capacity in primary fibroblasts upon serial sub-cultivation in culture [6]. In cardiovascular systems, fibroblasts are considered as the most abundant cell populations. In response to stresses, the cardiac fibroblasts usually undergo a phenotypic transformation into myofibroblasts and regulate α-SMA and extracellular matrix components in heart. With stressors that persisted, the cardiac fibroblast developed irreversible senescence with increased cell cycle proteins and SASP expression. Recent studies reported that the expression of senescence biomarkers, including p16 and p21, were remarkably increased in mouse heart post myocardial infarction [50,80]. Costaining of α-SMA (myofibroblast marker) with p53 or p16 confirmed increased senescent fibroblasts in infarct-border regions [50]. Likewise, Meyer and colleagues detected fibroblast senescence in two murine models with cardiac hypertrophy and remodeling [58]. As expected, the senescence biomarkers, including SA-β-gal, p16 and p21, were significantly increased in transverse aortic constriction (TAC) mouse model and β1-adrenoceptor (ADRB1) transgenic (TG) model. Dual staining of p21 and fibroblast markers (vimentin, platelet-derived growth factor receptor α or α-SMA) indicated that the senescent cells during cardiac remodeling are fibroblasts and myofibroblasts [58]. Significantly, these senescent cells were accumulated within fibrotic areas. Considering the pivotal role of senescence in injury repairing, recent studies have determined the presence of senescent fibroblasts during neonatal heart resection [51,81]. Feng et al used *Periostin-CreER*; *R26-loxp-stp-loxp-tdTomato* mice to label myofibroblasts as the periostin is highly expressed in activated fibroblasts [81]. The *dTomato*^+^*CDKN1A*^+^ myofibroblasts were identified in the peri-resected regions after apical resection at P3, P7 and P14. At the same time, Sarig also proved the presence of senescent fibroblast in injured P1 hearts by the dual-staining of SA-β-gal activity and vimentin [51]. Collectively, the senescent fibroblast/ myofibroblasts exist within the fibrotic areas ubiquitously and involved in fibrotic-related myocardial pathologies.

#### 3.2.5T cell senescence

During chronic aging, the immune system usually experiences prominent alterations that have fundamental impacts on health and survival. A wide range of age-related diseases shared an inflammaging pathogenesis [82]. One of the most important characteristics of immunosenescence was the age-dependent accumulation of memory T cells and reduction of naïve T cells in the immunological space. CD28, primarily expressed by naïve T cells, is a major costimulatory receptor that mediates T cells activation and proliferation [83]. Yet, the peripheral blood T cells become CD28^-^ during aging as the exhaustion of naïve T cells after lifelong chronic antigenic stimulation [84,85]. This stress-induced progressive loss of CD28 expression in T cells is accompanied with telomere shortening and cell cycle arrest [84,86]. It is important to note that CD28^-^ T cells play crucial role in age-related cardiovascular diseases. Spyridopoulos et al found accelerated telomere shortening in CD4^+^CD28^-^ T cells isolated from patients with coronary heart diseases [87]. Cytomegalovirus (CMV) is a well-known antigen that involved in immune-senescence. The CMV-positive CD8^+^CD28^-^ T cells are characterized with shorter telomeres. As expected, the CMV-seropositive patients showed a higher CD4^+^CD28^-^ T cells compared with the control patients with coronary diseases [87]. Therefore, the loss of CD28 was one of the most profound changes in T cells during immune aging. The gain of CD57 receptor, which occurs during the later stage of CD28^-^ T cell differentiation, was also defined as a distinct marker of replicative senescent T cells [88]. Consistent with CD28^-^ T cells, the CD57^+^ T cells were characterized with decreased proliferation capacity and increased pro-inflammatory cytokines during aging [89,90]. Both CD28^-^ and CD57^+^ T cells are considered as replicative senescent T cells in humans and the frequency of CD28^-^ or CD57^+^ T cells are increased in the peripheral blood with age [91,92]. For example, the relative frequency of immunosenescent CD28^-^ or CD57^+^ T cells were increased in patients with hypertension [93]. Additionally, CD57^+^ T cells have been shown to accumulate preferentially in the peripheral blood after acute myocardial infarction and acute failure, which correlated with the clinical outcome in patients with acute myocardial infarction and heart failure [90,94]. Therefore, the senescent T cells are involved in various cardiovascular diseases and further investigation of the mechanisms by which senescent T cells contribute to cardiovascular disorders is warranted.

## 4Consequence of cellular senescence in cardiovascular diseases

**Figure 4.**
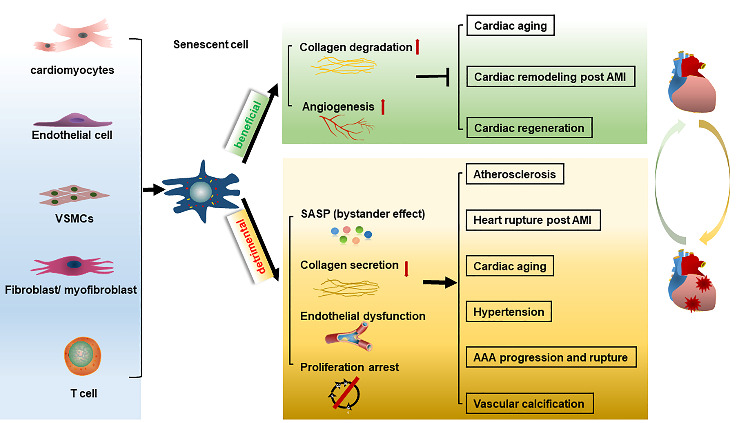
The pathological processes of cellular senescence in cardiovascular diseases. The senescent cardiovascular cells exert both beneficial and detrimental effect in cardiovascular diseases in a context-dependent manner. On the on hand, the senescent cells can restrict the fibrosis and prevent cardiac remodeling during ageing-related cardiovascular diseases. Moreover, senescence-associated secretory phenotypes (SASP) can modulate inflammation and promote angiogenesis during cardiac regeneration and myocardial infarction by bystander effects, which contributing to decreased cardiac dysfunction. On the other hand, the senescence is proposed as a key negative mechanism in promoting various age-related cardiovascular diseases (including atherosclerosis, heart rupture post AMI, cardiac aging, abdominal aortic aneurysm (AAA) and hypertension).

Key questions remain regarding the biological function of cellular senescence in cardiovascular system. Therefore, the clinical consequence of senescent cells in cardiovascular diseases is highlighted here and is classified as beneficial or detrimental effect in a context-dependent manner (Fig. 4).

### 4.1 Beneficial effects

Cellular senescence is known as a potent anti-tumor mechanism by stalling tumor cell proliferation. The preliminary results from phase I and II clinical trials of azidothymidine, a telomerase inhibitor, suggested a certain regression efficiency in solid tumors [95]. Despite withdrawing from cell cycle, the senescent cells remain viable and metabolically active. Over time, programmed senescence expressed an anti-fibrotic genetic program and restricted fibrosis during embryonic development and tissue repairing. For example, inducing cutaneous wounds in mice resulted in an accumulation of senescent cells in the granulation tissue of healing wounds, which protected against excessive fibrosis during wound healing [96].

#### 4.1.1 Anti-fibrotic effect in cardiac remodeling

Diffuse myocardial fibrosis is a pathological hallmark of most cardiovascular diseases that alters myocardial architecture and impairs cardiac function. Recent studies have uncovered a critical role of premature senescence in cardiac remodeling according to the activated SASP. Notably, the senescent cells downregulate genes encoding extracellular matrix components and upregulate degrading enzymes (including MMP). Such biological consequences may be broadly relevant to restrain fibrosis. As expected, CCN1 overexpression elicited senescent myofibroblasts accumulation in the fibrotic areas and limited fibrosis progression after TAC [58]. Conversely, genetic disruption of *Trp53^-/-^;Cdkn2α^-/-^* was associated with aggravated fibrosis and cardiac dysfunction after TAC [58]. Such loss-of and gain-of-function models suggested that senescent fibroblasts prevented excessive fibrosis during chronic pressure overload.

#### 4.1.2 Senescent fibroblasts protect against MI

Following acute MI, the sudden reduction or interruption of coronary blood flow may contribute to myocardial death. Angiogenesis can improve myocardial cell survival and preserve heart function following acute MI. Studies have shown that SASP-related inflammatory cytokines can promote the formation of new capillaries by sprouting preexisting vessels [97]. Of note, genetic inactivation of senescence by ATM haplodeficiency inhibited angiogenesis and endothelial tubing formation in response to acute MI, which accelerated heart failure in vivo [80]. Similarly, Shibamoto et al observed that blockade of DDR activation sustained CF proliferation and exacerbated cardiac fibrosis after MI [98]. Therefore, the senescent fibroblasts promoted collateral angiogenesis and limited cardiac fibrosis at the early stage following MI. Persistent myocardial ischemia is correlated with ventricular remodeling and cardiac dysfunction. Notably, in line with the anti-fibrotic role of senescence, ATM haplodeficiency aggravated the fibrotic area and cardiac hypertrophy during the chronic phase post-MI remodeling [80]. Despite the mechanisms differed between acute and chronic cardiac injuries, it is accepted that senescent fibroblasts can restrict fibrosis during chronic remodeling and promote angiogenesis during acute phase.

#### 4.1.3 Promoting cardiac regeneration

Previous studies proposed a crucial role of senescence in organismal regeneration and development. In this regard, many studies have pointed out the importance of senescent fibroblast in cardiac regeneration post neonatal heart resection [51,81]. Feng demonstrated that clearance of senescence by ABT263 treatment or *Trp53* knockout significantly aggravated fibrosis and inhibited neonatal cardiac regeneration [81]. Whereas CCN1 overexpression accelerated senescence in the injured area and promoted regeneration in neonatal mammalian heart. In addition, the SASP components can promote neonatal cardiomyocytes proliferation in vitro. Therefore, cellular senescence is required for neonatal heart regeneration, which is probably mediated by SASP-related angiogenesis and inflammatory response at the site of injury.

#### 4.1.4 Beneficial role of senescent cardiomyocytes

The cells within the myocardial microenvironment communicate with each other to maintain cardiac homeostasis. In addition to the senescent fibroblasts, cardiomyocytes senescence can also exert anti-fibrotic effects and regulate the surrounding cells via activating SASP complex. For example, Cui et al demonstrated that the senescent cardiomyocytes improved heart function via activating GATA4-dependent CCN1 secretion in ischemic heart [56]. CCN1 is an important component of SASP, which can restrict fibrosis and improve cardiac dysfunction postinfarction. Specifically, GATA4 knockdown arrested CCN1 secretion and aggravated heart dysfunction after MI. Therefore, cardiomyocytes senescence was necessary to prevent post-acute MI pathological fibrosis and heart dysfunction. Promoting senescence may be a potential mean in promoting neonatal heart regeneration and improving cardiac remodeling. Further understanding of how to employ these beneficial effects of senescence may provide novel target in preventing cardiovascular diseases.

### 4.2 Detrimental effects

Although cellular senescence favors organism remodeling and wounds healing as described above. Compelling data indicated that the persistent senescence during aging also linked with various pathological processes such as inflammation, oxidative stress, insulin resistance, metabolic disorder and loss of cell function [99]. Given its distinct role in each of these processes, cellular senescence is proposed as a key negative mechanism in promoting cardiovascular diseases.

#### 4.2.1 Atherosclerosis

Atherosclerotic dysregulation is characterized by the formation of fibro-fatty lesions in the intima of arteries, which is associated with dysregulation of endothelial cells, VSMCs and foamy macrophages. Senescent-like endothelial cells, VSMCs and foamy macrophages are obviously present in atherosclerotic plaques, and contribute to plaque progression, remodeling and stabilization. For example, the adhesion molecules and pro-inflammatory cytokines were increased in senescent ECs and damaged endothelial function during the development of atherosclerosis [17]. Elimination of endothelial senescent cells by *angptl2* knockdown promoted endothelium repair and limited the progression of atherosclerotic lesions in aortas wall [68]. Moreover, the bioavailability of NO was limited in senescent cells, which usually accompanied with disturbed redox balance and endothelial dysfunction. A recent study has verified an NO-dependent mechanism to delay endothelial cell senescence, thereby reducing the coronary risk factor-related atherosclerosis [100]. These studies verified that senescent ECs promoted atherosclerosis progression via complex mechanisms.

The most serious complication of atherosclerosis arises from the rupture of atherosclerotic plaques, which is largely correlated with structure of fibrous cap in vulnerable lesion. VSMCs in fibrous cap may undergo senescence and promote plaque instability by producing inflammatory adhesion molecules and matrix metalloproteinase (reviewed in Ref [70,72]). A recent study has recognized the role of senescent VSMCs in atherosclerosis by interfering with Sirt6. Grootaert et al demonstrated that Sirt6 delayed VSMCs senescence by preserving telomere integrity, thereby reducing atherosclerotic plaque burden and preserve plaque stability [101]. Therefore, the senescent VSMCs that accumulated in the atherosclerotic plaque could enhance its susceptibility to rupture. Additionally, vascular calcification is also associated with a significant increase in atherosclerotic plaque rupture. VSMCs senescence has been found to be the main contributor of vascular calcification by activating the expression of bone-related genes, including osteopontin [102]. Therefore, VSMCs senescence can accelerate atherosclerotic plaque rupture by promoting vascular calcification.

Histological examination of the early atherosclerotic lesion by transmission electron microscopy revealed a high accumulation of senescent foamy macrophages in the fatty-streak lesions, accompanied with intact elastic fibers and no fibrous cap. However, the sub-endothelial senescent foamy macrophages could express VCAM1 and MCP1 to recruit circulating monocyte and further promoted the development of senescent foamy macrophages, resulting in multiple inflammatory cytokines and MMPs. Over time, the senescent foam cells in atherosclerosis-prone *Ldlr^-/-^* mice drove elastic fiber degradation, fibrous cap thinning and plaque instability at the late stage of atherogenesis [103]. Indeed, atherosclerosis is a complex pathological process that can be regulated by different senescent cells in heart.

#### 4.2.2 MI

Acute myocardial infarction is the leading cause of morbidity and mortality worldwide. The degree of myocardial infarction is closely associated with cardiomyocytes loss and pathological remodeling with immune cells infiltration and fibroblasts activation in the cardiac extracellular matrix. Following myocardial infarction, inhibition collagen deposition may have a complementary role in restricting pathological remodeling, however, it remains crucial to provide mechanical strength and prevent cardiac rupture. Zhu et al demonstrated that P53-mediated fibroblast senescence restricted collagen deposition and cardiac fibrosis, which resulting in cardiac rupture and cardiac dysfunction after myocardial infarction [50]. Conversely, knockdown of P53 inhibited fibroblast senescence and enhanced collagen deposition and reparative fibrosis postinfarction. There was, however, a contradictory result from the same lab demonstrated that depressing senescence by ATM deficiency impaired angiogenesis and accelerated heart failure in response to MI [80]. One potential explanation is that the MI is a dynamic and complex processes involved in different cells. Memory T cell populations usually undergo senescence and exert pro-inflammatory and high cytotoxic capacities postinfarction. The immunophenotyping revealed a link between the frequency of senescent CD8+CD57+ T cells and short-term cardiovascular mortality in patients following acute myocardial infarction [94].

#### 4.2.3 Cardiovascular aging

During aging, the cardiomyocytes contractility is impaired due to the maladaptation of cellular metabolism and stressors. The senescent cardiomyocytes showed hallmarks of DNA damage, mitochondrial dysfunction, hypertrophic growth, contractile disorder and SASP [29,104]. According to a recent review, the communications between senescent cardiomyocytes and non-myocytes within the myocardial microenvironment were clearly discussed [61]. Upon stress, senescent cardiomyocytes modulated microenvironment to maintain functional compensatory response or decompensatory collagen deposition and remodeling. Although activation of senescent fibroblasts was directly linked with fibrosis restriction during physiological cardiac aging [105]. The senescent-like cardiomyocytes isolated from aged mice may activate a number of senescence pathways with pro-fibrotic and pro-hypertrophic capacities via a non-typical SASP [29]. The conditioned medium isolated from aged cardiomyocytes induce α-SMA expression in myofibroblast, such bystander effect may partly explain the detrimental role of senescent cardiomyocytes in cardiac aging. However, the understanding of the microenvironmental communication between senescent cells and non-senescent cells is still poor.

Aging-related vascular disorders are primary presented by arterial stiffness and calcification [106,107]. Recently, Regnault has reviewed the relationship between vascular aging and vascular cell senescence [106]. They summarized that the senescent VSMCs exhibited increased expression of bone-related genes and adopt a specific calcifying phenotype during aging. Specifically, Liu has demonstrated the implication of VSMCs senescence in vascular calcification by disturbing lamin A [102]. Continuous DNA damage by Prelamin A overexpression accelerated VSMCs senescence and developed an osteogenic phenotype subsequently. The specific SASP factors, including BMPs, Il-1β, Il-6 and osteoprotegerin, were abundantly increased in senescent VSMCs and correlated with vascular dysfunction in aged animal models and aged humans. Therefore, targeting senescent vascular cells may be potential therapies for age-related vascular calcification.

#### 4.2.4 AAA

AAA is an age-related vascular disease that develops asymptomatically until rupture occurs. The pathophysiology of AAA is highly complex, with elastic tissue degeneration, inflammation, VSMCs dysfunction and accelerated oxidative stress [107]. More importantly, recent studies have linked cellular senescence to AAA pathogenesis [75-77,108]. SIRT1 inhibition accelerated vascular cell senescence and facilitated the transcriptional activation of nuclear factor-κB (NF-κB) signaling, which promoted the formation and rupture of AAAs [75]. Whereas genetic activation of SIRT1 inhibited p21-dependent senescence and suppressed AAA formation and progression. Although the precise mechanisms underlying VSMCs senescence in AAA remain unclear. The senescent VSMCs released a variety of pro-inflammatory cytokines and matrix-degrading molecules, which critically correlated with AAA progression. More recently, great progress has been made in AAA treatment by interfering VSMCs senescence. Gao et al demonstrated that myocardin related transcription factor A deficiency suppressed AngII-related AAA progression by decreasing VSMCs senescence [77]. Therefore, VSMCs senescence may provide an alternative strategy for AAA prevention.

#### 4.2.5 Others

Early studies have shown that the immunosenescent T cells are accumulated in individuals with normal aging and impair the adaptive immune system in the elderly [83]. There are immunologic, serological and histological evidence to demonstrate the connection between immunosenescent T cells and hypertension in humans [93]. The pro-inflammatory senescent T cells release cytokines and result in arteriolar remodeling during the progression of hypertension. Moreover, cytomegalovirus specific senescent CD8+CD57+ T cells are independently correlated with age-related arterial stiffness and cardiac dysfunction [92]. Taken together, abundance of transgenic and pharmacological approaches demonstrated a detrimental role of senescent cells in cardiovascular disorders, which weak the repair of the cardiac damage.

## 5Factors regulating senescence in cardiovascular diseases

The primary molecular regulators of lifespan are SIRT and mammalian target of rapamycin (mTOR). Each of them plays central role in modulating senescence and cardiovascular diseases (Table 2).

**Table 2:** The factors regulating senescence in cardiovascular diseases.

Targets	Ref	Model	Related senescent cells	Expression /activity	Mechanisms regulated by SIRT1	Significance
SIRT1	[166]	In vitro	ECs-AngII/H2O2	↓	N	SIRT1 activation alleviated vascular ECs senescence
	[117]	In vitro	CMs-Doxorubicin	↓	N	Roflumilast alleviated CMs senescence by activating SIRT1
	[118]	In vitro	ECs-AngII	↓	N	SIRT1 activation alleviated AngII-induced ECs senescence
	[167]	In vitro	ECs-AngII	↓	N	Resveratrol alleviated AngII-induced senescence by activating SIRT1
	[168]	In vitro	ECs-replicative /H2O2	↓	N	FGF21 alleviated endothelial senescence by activating SIRT1
	[47]	In vitro	ECs-Sirtinol	↓	AC-p53↑	SIRT1 activation prevented stress-induced senescence in ECs
	[119]	In vivo	VSMCs-AAA	↓	AC-p53 ↑	SIRT1 activation alleviated vascular senescence in AAA
	[123]	In vitro	ECs-high glucose	↓	AC-p53/FOXO1 ↑	Metformin alleviated endothelial senescence by activating SIRT1
	[169]	In vitro	ECs-replicative senescence	↓	AC-FOXO1 ↑	SIRT1 inhibition by miR-217 promoted endothelial senescence
	[128]	In vitro	VSMCs-Doxorubicin	↓	AMPK (Ser485) ↓	SIRT1 activation alleviated VSMCs senescence
	[127]	In vitro	VSMCs-Doxorubicin	↓	AMPK (Ser485) ↓	Prednisolone prevented VSMCs senescence by activating SIRT1
	[124]	In vitro	ECs-replicative senescence	↓	AMPK (Thr172) ↑	SIRT1 protected against endothelial senescence via AMPK pathway
	[170]	In vivo	ECs-AS	↓	SIRT1-LKB1	Roscovitine alleviated senescence by inhibiting SIRT1 phosphorylation
mTOR	[171]	In vitro	ECs-replicative senescence	N	N	Rapamycin alleviated ECs senescence
	[172]	In vitro	VSMCs-AngII	↑	N	Hyperactivation of mTOR by AngII elicited senescence
	[132]	In vivo	Vascular senescence-Obesity	↑	N	Rapamycin alleviated vascular senescence by inhibiting mTOR
	[135]	In vivo	Cardiac senescence-aging	↑	Autophagy ↓	Caloric restriction alleviated senescence by inhibiting mTOR
	[133]	In vitro	VSMCs-oxLDL	↑	Autophagy ↓	Rapamycin alleviated VSMCs senescence by inhibiting mTOR
	[134]	In vitro	VSMCs-Adriamycin	↑	Autophagy ↓	Rapamycin alleviated VSMCS senescence by inhibiting mTOR
	[173]	In vitro	CMs-aging	↑	Mitophagy ↓	Rapamycin stimulated mitophagy and alleviates senescence

ECs, endothelial cells; AngII, angiotensin II; CMs, cardiomyocytes; VSMCs, vascular smooth muscle cells; AAA, abdominal aortic aneurysm; AS, atherosclerosis; MI, myocardial infarction; FGF21, fibroblast growth factor 2; AC, acetylation; N, not mentioned.

### 5.1 SIRT

SIRT are an evolutionarily conserved family of (nicotinamide adenine dinucleotide) NAD+-dependent protein lysine deacylases that play critical role in longevity and health aging [109]. Among mammalian SIRT subtypes, SIRT1 is a well-recognized class III histone deacetylase that deacetylates various downstream transcription factors, including p53, forkhead transcription factors (FOXO) and nuclear factor κB (NF-κB) [110,111]. Therefore, SIRT1 has been identified as a longevity gene by regulating cell cycle, inflammation, oxidative stress, metabolism and premature senescence in mammalian cells [112,113]. A recent study has summarized a therapeutic role of SIRT1 in preventing aging-related organ fibrosis, including heart [114]. During cardiac aging, the SIRT1 protein is downregulated. The decreased SIRT1 expression and activity were closely associated with the progression of cellular senescence and cardiac dysfunction [115]. Conversely, SIRT1 activation confers protective effects on cellular senescence involving cardiovascular system [116-118]. Although the mechanisms remain unclear, SIRT1 inhibition-mediated p53 acetylation was implicated in aging-related cardiovascular disorders. Ota et al demonstrated that SIRT1 expression was reduced in ECs obtained from aged mouse aortas, and SIRT1 deficiency promoted senescence by enhancing p53 acetylation in ECs [47]. Whereas overexpression of SIRT1 antagonized hydrogen peroxide-induced ECs senescence. In vivo, SIRT1 expression and activity were significantly decreased in AAA tissues, accompanied with upregulated p53 acetylation and p21 expression [119]. VSMCs-specific overexpression of SIRT1 inhibited p21-dependent VSMCs senescence and arrested AngII-induced AAA formation and rupture in mice.

Notably, p53 is not the primary SIRT1 deacetylation substrate in heart. SIRT1-dependent FoxO1 deacetylation was also involved in stress-related senescence, angiogenesis, oxidative stress and apoptosis [120,121]. The increased FOXO1 acetylation may result in the switching of FOXO1 activity towards transcription of cell cycle arrest genes [122]. HUVECs senescence by SIRT1 inhibition was associated with increased FOXO1 acetylation and endothelial dysfunction. Exposure of ECs to high glucose remarkably reduced SIRT1 expression, concomitant with accelerated FOXO-1/p53 acetylation and increased p21 expression [123]. Therefore, SIRT1 downstream targets FOXO1 and p53 link SIRT1 inhibition and senescence in cardiovascular diseases. The crosstalk between SIRT1 and AMPK also plays a central role in senescence. Generally, AMPK is activated by phosphorylation in the presence of an elevated AMP/ATP at Thr172. SIRT1 inhibition-dependent LKB1/AMPK (Thr172) hyper-activation was implicated in endothelial senescence [124]. Although the phosphorylation of AMPK (Thr172) is the primary phosphorylation site that activates AMPK, phosphorylation of AMPK at Ser485 by AKT and PKA pathway is believed to have an inhibitory effect on AMPK activation [125,126]. Sung et al demonstrated that during Adriamycin-induced VSMCs senescence, the SIRT1 expression and AMPK (Ser485) phosphorylation were significantly deceased [127,128]. SIRT1 activation by SRT1720 or Prednisolone suppressed Adriamycin-induced VSMCs senescence by activating cAMP-PKA/p-AMPK (Ser485) pathway [127,128]. Therefore, manipulation of SIRT1 activation may effectively attenuate senescence and prevent age-related cardiovascular diseases by interfering different downstream targets.

### 5.2 mTOR

mTOR is a highly conserved serine/threonine protein kinase that regulates growth-related processes, including protein synthesis, cell proliferation and autophagy [129,130]. Previous studies have demonstrated that pharmacological and genetic inhibition of mTOR extended lifespan and delayed the pathogenesis of aging-related organism dysfunction effectively [131]. In the setting of advancing aging, a significant activation of mTOR signaling was observed and was claimed to be a key molecule in switching on/off senescence. Recent studies have demonstrated that sustained mTOR signaling activation linked obesity with vascular senescence and cardiovascular diseases [132]. Rapamycin treatment prevented vascular senescence and reduced the severity of ischemic strike [132]. Moreover, hyperactivation of mTOR by arginase-II resulted in VSMCs senescence. Although the mechanisms remain unclear, mTOR activation-related autophagy depression is involved in senescence and cardiovascular diseases.

Autophagy is a cellular housekeeping mechanism that helps maintain homeostasis by degrading damaged protein and organelles. Inadequate autophagy is associated with increased oxidative stress, inflammation and cellular dysfunction. Therefore, mTOR-dependent autophagy inhibition appears to promote aging and age-associated diseases by modulating premature senescence. For example, Luo et al demonstrated that mTOR inhibitor Rapamycin significantly reduced the expression of senescence marker p21 and p16 in senescent VSMCs by regulating mTOR/ULK1-dependent autophagy [133]. In contrast, autophagy inhibition by 3-MA accelerated VMSCs senescence and promoted atherosclerosis progression. More recently, Sung et al found that Adriamycin-induced VSMCs senescence was accompanies with increased activity of mTOR [134]. Pharmacological inhibition of mTOR signaling cascade significantly decreased VSMCs senescence. Caloric restriction (CR) is a well-established intervention in preventing age-related disorders. Notably, a recent study proved that CR conferred cardioprotection in aged mice by retarding cardiac senescence [135]. A possible mechanism by which is the enhanced autophagy with suppressed mTOR signaling during long-term CR. These results demonstrated that the mTOR-autophagy signaling might be critical in regulating senescence-associated cardiovascular diseases. Enhanced autophagy by mTOR inhibition is considered to be protective in senescence by degrading damaged proteins and organelles.

Recently, a positive relationship between mTOR activity and SASP has been suggested in senescent cells [136,137]. Using the mTOR inhibitor, rapamycin, Laberge et al found that the secretion of SASP components in senescent fibroblasts are mTOR-dependent [138]. Specifically, the pro-inflammatory cytokines were significantly attenuated by rapamycin in the context of senescence. Although, the mTOR regulation of SASP has not been studied in cardiovascular system nowadays. The discovery of the anti-SASP activity of mTOR inhibition is encouraging and provides a rationale for the anti-senescent therapy in the near future.

**Table 3:** Senolytics treatment in age-related cardiovascular diseases.

Senolytics	Cardiovascular diseases	Dosage	Significance	Ref
D+Q	AS	Q(20mg/kg/d) for 8 weeks	Q eliminated senescent cells and alleviated AS lesions	[146]
	Aged heart	D(5mg/kg)+Q(50mg/kg) for a single dose	D+Q eliminated senescent cells and alleviated age-related cardiac dysfunction	[147]
	Aged heart	D(5mg/kg)+Q(50mg/kg) for 3 days every 3 weeks for 2 months	D+Q eliminated senescent cells and alleviated age-related cardiac dysfunction	[149]
	Aged heart/AS	D(5mg/kg)+Q(10mg/kg) once/month for 3months	D+Q eliminated senescent cells and alleviated age-related vascular disorders	[148]
Navitoclax (ABT263)	AS	100 mg/kg for a single dose	ABT263 eliminated senescent cells and reduced atherogenesis onset	[103]
	I/R	50mg/kg/d for 7 days	ABT263 eliminated senescent cells and improved cardiac function following I/R	[155]
	Aged heart	50mg/kg/d for 7 days a cycle for 2 cycles with a 1-week internal between cycle	ABT263 eliminated senescent cells and reduced hypertrophy in aged mice	[29]
	Aged heart/ MI	50mg/kg/d for 7 days a cycle for 2 cycles with a 1-week internal between cycle	ABT263 alleviated senescence in aged mice and improved heart function following MI	? [153]
	AngII	50mg/kg/d for 7 days a cycle for 2 cycles with a 1-week internal between cycle	ABT263 eliminated senescent cells and improved cardiac dysfunction induced by AngII	[154]
	PAH	10mg/kg/d for 7 days	ABT263 eliminated senescent cells and reversed established PAH	[159]

D, dasatinib; Q, quercetin; AngII, angiotensin II; PAH, pulmonary arterial hypertension; I/R, ischemia/reperfusion injury; MI, myocardial infarction; AS, atherosclerosis.

## 6.Targeting senescence for treatment of cardiovascular diseases

Cellular senescence has emerged as an attractive target in preventing age-related cardiovascular disorders [9] [9] [9]. Therapeutical approaches including pro-senescent and anti-senescent are well studied in several areas of aging pathology [139]. Elimination of vascular endothelial senescent cells stimulated endothelial repair and delayed the progression of atherosclerosis in mice [68]. In 2011, Baker and his colleagues established a novel transgene, *INK-ATTAC*, to induce specific apoptosis in p16^Ink4α^-positive senescent cells upon AP20187 administration [140]. Additionally, the *p16-3MR* mice were also engineered to kill p16^Ink4α^-expressing senescent cells with ganciclovir or metronidazole administration through distinct mechanisms [141]. These highly influential works are foundations of anti-senescent therapy in animals. For example, elimination of p16^Ink4α^-positive senescent cells in aged mice alleviated the myocardial hypertrophy and fibrosis during cardiac aging [29]. Additionally, clearance of senescent cells by ganciclovir stabilized the fibrosis cap in atherosclerosis and showed no toxic side effect [11]. The striking improvement in genetic mice largely promotes the development of pharmaceutical approaches to eliminate senescent cells in vivo. Senotherapeutics are a class of drugs that function to eliminate senescent cell (senolytics) and suppress cell-extrinsic effect of senescent cells (senomorphics/senotatics) [4,142]. Here, we will discuss the most recognized senolytics in ageing-related cardiovascular diseases (Table 3).

Clearing senescent cells extends lifespan in mice. Yet, the senescent cells are highly resistant to apoptosis owing to the increased expression of pro-survival B cell lymphoma-2 (BCL-2) family proteins (BCL-2, BCL-W, and BCL-XL) [143]. Various senolysis strategies have been developed to eliminate senescent cells by targeting anti-apoptotic signaling molecules in the context of aging [142-144]. For example, dasatinib (D) facilitates apoptosis of senescent cells by inhibiting ephrins receptors-dependent tyrosine kinase. Quercetin (Q) is a natural flavonoid that induce apoptosis in senescent cells by inhibiting PI3K/mTOR signaling [145]. Jiang et al found that quercetin alleviated atherosclerotic lesions and effectively protected against oxidized LDL-related endothelial senescence in aorta of *ApoE*^-/-^ mice [146]. Based on their known targets, the combination of quercetin and dasatinib is more effective in clearing senescence in different pathological models. Five days after a single dose of D+Q, Zhu demonstrated that senolytics selectively ablated senescent cells and improved cardiac and vascular function in aged mice [147]. Moreover, D+Q treatment had a persisting phenotypic effect 7 months following drugs treatment. Yet, the accumulation of senescent cells is a dynamic and continuous process, intermittent treatment with D+Q appears to be more effective than a single dose. Roos and collages treated aged mice with D (5mg/kg) +Q (10mg/kg) once monthly for 3 months [148]. Notably, they found that chronic senolytic treatment alleviated TAF-positive senescent cells in the medial layer of aorta from aged and atherosclerotic mice, accompanied with improved vasomotor function in hypercholesterolemia-induced vascular pathology. Additionally, intermittent oral administration of D (5mg/kg) +Q (50mg/kg) for three consecutive days every 2 weeks for 2 months restored aging-related cardiac dysfunction and rejuvenated cardiac regenerative capacity [149]. The remarkable advantage of using a single dose or intermittent administration is that the side effects would be less common compared with continuous senolytics treatment. However, it should be note that high dose of dasatinib was shown to induce DNA damage and exacerbated the development of pulmonary arterial hypertension in rat [150,151].

Navitoclax, otherwise known as ATB-263, is a potent inducer of apoptosis by inhibiting Bcl-2 family proteins [152]. Previous studies have shown that ABT263 have a direct role in age-related cardiomyopathy. Removal of senescent cells by ABT263 significantly reduced age-dependent cardiac remodeling and improved cardiac function and survival outcome following MI [153]. Similarly, ABT263 attenuated atherogenesis onset in *Ldlr*^-/-^ mice by selectively clearing senescent foamy macrophages [103]. The prevalence of heart failure dramatically increases in aged populations. In contrast to the observation that ABT263 has no significant impact on cardiac function in aged mice [153]. Jia et al demonstrated that ABT263 dramatically improved cardiac dysfunction and decreased cardiac fibrosis, hypertrophy and inflammation in Ang II-infused mice [154]. A key component of cardiac ischemia-reperfusion injury is the increased accumulation of senescent cells, resulting in adverse ventricular remodeling and impaired cardiac function. Dookun et al demonstrated that ABT263 treatment cleared senescent cells and improved cardiac function following cardiac ischemia/reperfusion injury [155]. Collectively, removal of senescent cells by ABT263 delays the onset and development of cardiovascular diseases, including atherosclerosis, myocardial infarction, ischemia-reperfusion injury and cardiac aging [29,103,153,155].

A recent study demonstrated that D+Q has already tested in clinical settings of idiopathic pulmonary fibrosis-related dysfunction, which suggesting the anti-senescent agents may be within reach in human. Thus, it is worthwhile to optimize the anti-senescent protocols for clinical translation in the near future.

## 7. Conclusion and perspective

Cellular senescence is becoming increasingly recognized as a therapeutic target in ageing-related cardiovascular pathophysiology [13,156]. On the one hand, cellular senescence provides clear benefits in cardiac remodeling following multiple pathological conditions, including myocardial infarction, pressure-overload related hypertrophy and cardiac aging. On the other hand, many proof-of-concept evidence suggests that clearance of senescent cells with genetic and pharmacological methods are developing strategies to revers age-related cardiovascular diseases. Currently, some challenging are still exist and are urgently to be solved in future researches and clinical trials. Firstly, the lack of specific biomarkers for senescent cardiovascular cells makes it difficult to identify the special senescent cell in vivo. We need to uncover unique cellular senescence markers in cardiovascular diseases. Secondly, it is critically important to explore cell-specific senolytic compounds that act on special senescent cardiovascular cell types without side effects. Thirdly, the beneficial effect of cellular senescence, including pro-angiogenesis, anti-cancer and anti-fibrotic, would limit the effective therapeutic window for senolytic compounds. Finally, great attention should be paid to optimize the dosage and courses of senolytic treatment in preclinical studies. Inappropriate dosage may either weaken the therapeutic efficiency or damage health cells. As therapeutic exploitation by targeting cellular senescence might be possible, understanding the diverse roles and mechanisms of cellular senescence in cardiovascular diseases helps develop novel agents and determine appropriate clinical strategies.
